# Can we predict postoperative dysphagia after anterior cervical discectomy and fusion based on lateral x-ray? An external validation of two outcome scores

**DOI:** 10.1007/s10143-026-04142-y

**Published:** 2026-02-17

**Authors:** Gloria Cabrera, Andrés Rojas-Gil, Nuria Montagut, Jorge Herrero Hernando, Jorge Torales, Alberto Di Somma, Abel Ferrés, Pedro Roldán, Ramon Torné, Alejandra Mosteiro, Jose Poblete Carizo

**Affiliations:** 1 Department of Neurosurgery, Hospital Clínic Barcelona, University of Barcelona, Barcelona, Spain; 2 Department of Surgery, Faculty of Medicine, University of Barcelona, Barcelona, Spain

**Keywords:** Dysphagia, ACDF, Cervical, Prediction, X-Ray

## Abstract

Postoperative dysphagia following Anterior cervical discectomy and fixation (ACDF) impacts quality of life and risks airway complications. Identification of retropharyngeal oedema with a lateral Xray has gained interest as screening method. We prospectively evaluated the relation between dysphagia and two radiological scores to determine the external predictive value. A prospective study (2021–2022) held in a tertiary centre, including a sequential sample of patients operated of ACDF for degenerative conditions. Postoperative (day + 1) evaluation of dysphagia was systematically performed by a speech and language therapist. The retropharyngeal swelling index (by Haws) and the dysphagia predicting score (by Yoshida) were calculated on lateral Xray. A logistic regression model and receiver operating characteristic (ROC) curves were calculated. 100 patients were included for analysis, of which 26% developed postoperative dysphagia (mild in 20% and mild-to-moderate in 6%). The most frequent form of dysphagia was oesophageal (18%), followed by oropharyngeal (4%) and pharyngoesophageal (3%). None of the potential risk factors studied were confirmed in our sample. For predicting oropharyngeal dysphagia, Yoshida score yielded an AUROC of 0.730 and the sweeling index an AUROC of 0.530. Conversely, in cases of oesophageal dysphagia, the AUROC were 0.590 and 0.504 respectively. From our data, it may be inferred that oropharyngeal and oesophageal dysphagia might harbour different underlying mechanisms, still not well understood. The use of a simple X-ray may become a routine screening step for patients undergoing ACDF. While it seems robust to predict oropharyngeal dysphagia, identification of oesophageal dysphagia may require further investigation.

## Introduction

 Anterior cervical discectomy and fixation (ACDF), first described by Smith, Robinson and Cloward in 1958 [1], is considered the gold standard for the treatment of cervical degenerative disc disease [2]. Besides its reasonable safety and low rates of morbidity, disabling complications may occur due to the proximity of key anatomical structures [3, 4]. Such is the case of the non-negligible rates of postoperative dysphagia [5].

Postoperative dysphagia impacts quality of life and poses a risk to developing airway complications, which can be potentially fatal [6]. The reported incidence rates of dysphagia after ACDF vary widely depending on the assessment methods, ranging from 17.5% to 71% [7–10]. Although most of the cases are self-limiting and resolve within 3 months [10], chronic dysphagia is reported in 3% to 35% of cases [10], affecting daily life and increasing health-related costs [6].

Yet, the aetiology of postoperative dysphagia remains inconclusive [3, 6], even when several risk factors have been identified [10–12]. This poses a challenge in terms of prevention, early identification and appropriate management. To this aim, identification of soft tissue oedema in the retropharyngeal space (RS) with a plain lateral Xray has gained interest as a simple screening method; indeed, two predictive scores have been described based on this finding [8, 13]. Their potential clinical utility is notable, however, the external validity of these scores has not been addressed.

In this prospective study we evaluated the relation between dysphagia after ACDF, and the two radiological scores based on changes in the RS to determine the external predictive value.

## Methods

### Study desing and sampling

This was a prospective study held in a single tertiary centre of reference for spinal degenerative disease. The sampling was sequential, and it included all adult patients operated for anterior cervical discectomy and fusion (ACDF) for degenerative conditions, including those with and without myelopathy. Patients were excluded from the study if preoperative dysphagia was present, or if the indication was traumatic or neoplastic pathologies. The studied period comprised from January 2021 to December 2022. All the interventions were performed by a team of specialist neurosurgeons, with at least five years of experience in cervical spine surgery. None of the patients received pre or postoperative steroids. The study protocol was approved by the institutional ethics committee (HCB/2023/1077), and it complied with the Declaration of Helsinki and with national and European regulations on biomedical research involving humans.

The prospective database included demographic variables and comorbidities, tabaco or alcohol consumption, previous cervical interventions, surgical indication, side of approach, number of cervical levels operated, duration of the intervention, length of hospital stay, and postoperative complications. Specifically, the incidence of postoperative dysphagia, odynophagia and dysphonia were recorded.

## Surgical protocol

All the interventions followed the same basic surgical steps, and the same instruments and retractors were used in all cases. Moreover, fusion was done with the same material in all cases, namely an intersomatic cage filled with hydroxyapatite (Nanogel) with trans-somatic fixation screws (Coalition MIS). An anterior plate was not used in any case. The side of surgical approach was chosen based on surgeon’s preference, being the right side the most frequently used. Intraoperative neurophysiologic monitoring was performed in cases with evidence of clinical or radiological myelopathy.

In brief, patients were positioned supine, with moderate neck hyperextension. A 3–4 cm lateral anterior horizontal cervical incision was performed, the neck was dissected between the vascular and the visceral triangles, until the prevertebral fascial was encountered. Cloward retractors were placed, with the valves inserted at the level of the longus colli muscle insertions. The anterior discectomy was performed, and the anterior and posterior osteophytes were removed with diamond drill. After adequate central canal and foramina decompression, an anterior cage was placed and fixed with trans-somatic screws. Intraoperative X-rays were systematically performed to confirm the adequate position of the cage. After cautious haemostasis, the wound was closed with simple stitches. A subcutaneous drain was not used.

Patients remained in the anaesthesia postoperative care for six hours, if no complications were detected, they were transferred to the conventional neurosurgical ward. Mobilization and physiotherapy started 8 h after the operation. No patient received postoperative steroids. Postoperative analgesia included a combination of non-steroid anti-inflammatory drugs and occasionally tramadol.

## Systematic evaluation of postoperative dysphagia

Postoperative evaluation of dysphagia, odynophagia and dysphonia was systematically performed by a speech and language therapist with specialised training in swallowing disorders, including formal training in the use of the Volume-Viscosity Swallow Test (V-VST) [14], and other standardized bedside screening procedures, as well as more than 10 years of clinical experience in the management of dysphagia. This combination of training and experience ensures consistent and reliable identification of postoperative swallowing impairments. The swallowing test was performed on postoperative day + 1 and repeated on day + 2 if the patient was still admitted in hospital. Discharged patients without dysphagia were instructed on warning symptoms and advised to contact speech and language therapy if these occurred, ensuring prompt evaluation of any delayed onset swallowing difficulties. Patients’ perception of swallowing was evaluated using the Eating Assessment Tool (EAT-10) [15]. Although widely used, the EAT-10 has known psychometric limitations, including item redundancy, floor effects, and suboptimal construct validity, as demonstrated by recent Rasch-based analyses [16]. For this reason, in our study the EAT-10 was used only to quantify the patient’s subjective perception of swallowing difficulty, while objective swallowing function was assessed separately using the V-VST, a standardized bedside screening tool for evaluating the safety and efficacy of swallowing across different volumes and viscosities.

In patients diagnosed with dysphagia, the disorder was further classified according to its anatomical location as oropharyngeal (difficulty initiating a swallow, coughing or choking), pharyngoesophageal (delayed bolus transit, sensation of sticking at the lower pharynx), or oesophageal (sensation of bolus sticking in the chest) dysphagia, based on the location of symptoms and bedside findings. This classification was selected because it reflects the anatomical regions most likely to be affected by anterior cervical surgery, particularly the oropharynx and the pharyngoesophageal segment, which are susceptible to surgical retraction and postoperative oedema. The category oesophageal was maintained to capture symptoms suggestive of distal involvement (e.g., sensation of bolus sticking, delayed transit), acknowledging that this classification is clinical and screening-based rather than pathophysiological definitive. The severity of dysphagia was determined using the Functional Oral Intake Scale (FOIS) [16], categorizing patients as having mild, moderate, or severe dysphagia. Mild dysphagia corresponded to FOIS levels 6–7 (mostly oral intake with minimal restrictions), moderate to levels 4–5 (partial oral intake with some modification or supplemental nutrition), and severe to levels 1–3 (limited or no oral intake, requiring tube feeding or complete dietary modification). This classification was applied to provide a clinically meaningful stratification of functional impairment, while acknowledging that FOIS was not originally designed to define severity categories [17, 18]. The oral intake was adapted with a specific diet according to the swallowing test results. In addition, patients were asked to rate the intensity of odynophagia on a 0–10 numerical rating scale, allowing differentiation between painful swallowing (odynophagia) and true swallowing difficulty (dysphagia) assessed with the EAT-10 and V-VST. The presence and severity of dysphonia were evaluated using the GRBAS scale (Grade, Roughness, Breathiness, Asthenia, Strain). Although perceptual and subject to inter- and intra-rater variability, the assessment was performed by a single experienced clinician to ensure consistency. While instrumental laryngoscope evaluation was not performed, the GRBAS provided a practical and clinically meaningful measure of postoperative voice changes. Moreover, patients diagnosed with dysphagia and/or dysphonia received a specific follow-up with speech and language therapy in the outpatient clinic until the symptoms resolved. Those with persistent dysphagia at 6 months underwent video fluoroscopic swallow study (VFSS), while patients with persistent dysphonia were referred to an otolaryngologist for further evaluation. Follow-up with speech therapy continued as indicated to ensure optimal functional recovery.

## Radiological evaluation of the retropharyngeal space

As part of the institutional protocol, patients undergo an anteroposterior and lateral plan Xray 6 h after the ACDF intervention to confirm the final position of the intersomatic cage. This lateral Xray was used in the present study to evaluate the retropharyngeal space and to calculate two specific dysphagia predicting scores.

The retropharyngeal swelling index, described by Haws et al.(8), was calculated as follows: Swelling index = AP diameter of prevertebral soft tissue/AP vertebral body x 100.

The dysphagia predicting score by Yoshida et al.(13) was calculated as follows: Distance (in mm) from the anterior surface of C2 inferior plate to the air column in the trachea.

### Statistical analysis

Statistical analyses were conducted using SPSS version 27 (IBM). When possible, variables were summarized as mean (standard deviation) or percentages. Continuous variables were checked for normality distribution using the Kolmogorov-Smirnov test. Then data were analysed using the Student t test, Mann-Whitney U test or Kruskal-Wallis test as appropriate. Categorical variables were analysed with the Pearson χ2 test. A *p*-value < 0.05 was defined as statistically significant. Variables showing *p*-value < 0.10 in univariate analysis were included in a multivariable analysis using a logistic regression model. Receiver operating characteristic (ROC) curves were constructed for both predictive scores.

## Results

### Patient characteristics

In the two-year period comprised between 2021 and 2022, a total of 102 patients were operated of ACDF in the authors’ institution. Of them, 2 were excluded because of preoperative dysphagia, so that 100 patients were included for final analysis. The clinical features are summarized in Table 1.

**Table 1:** Description of the sample. Clinical and radiological characteristics of the patients operated for anterior cervical discectomy and fusion (ACDF)

Variable	ACDF cohort (*n* = 100)
Age, mean (SD)	54 (12)
Sex, female, n (%)	52 (52%)
Comorbidities, n (%)	43 (43%)
Previous cervical surgery, n (%)	14 (14%)
Previous ACDF	10 (10%)
Other	4 (4%)
Body mass index, mean (SD)	26 (5)
Smoking, n (%)	42 (42%)
Alcohol, n (%)	3 (3%)
Surgical indication, n (%)	
Disc herniation	72 (72%)
Mielopathy	28 (28%)
No. of cervical levels, n (%)	
One	59 (59%)
Two	35 (35%)
Three or more	6 (6%)
Cervical level, n (%)	
Upper cervical (C3-C5), n (%)	1 (10%)
Lower cervical (C5-T1), n (%)	56 (56%)
Both, n (%)	34 (34%)
Side of intervention, right, n (%)	88 (88%)
Surgery durantion, min, mean (SD)	123 (49)
LoS, days, mean (SD)	3 (2)
Complications, n (%)	7 (7%)
Neurological deficit	3 (3%)
CSF fistula	2 (2%)
Cervical hematoma	1 (1%)

The mean age was 54 (± 12) years with almost equal gender distribution (52% female). The mean body mass index was 26 (± 5), about 42% of patients were smokers and 3% had alcohol dependency. Overall, 14 (14%) of the patients had undergone previous cervical surgery. The current indication included myelopathy in 28% of cases, and the number of levels operated were single in 59%, double in 35% and triple in 6%. The right side was the predominant approach (88%). Mean surgical duration was 123 (± 49) minutes and mean length of hospital stay was 3 days (± 2). Postoperative complications occurred in 7% of cases, namely: new neurological deficit (3%), cerebrospinal fluid fistula (2%) and cervical hematoma (1%).

## Swallowing outcomes and risk factors for postoperative dysphagia

Postoperative swallowing outcomes are summarized in Table 2. Concretely, 26% of patients developed postoperative dysphagia as evaluated on postoperative day + 1. In most cases, dysphagia was mild (20%), with only 6% mild to moderate. The most frequent form of dysphagia was oesophageal (18%), followed by oropharyngeal (4%) and pharyngoesophageal (3%). Besides, 56% of patients developed odynophagia and 22% dysphonia in the acute postoperative period.

**Table 2:** Swallowing outcomes

Outcomes	ACDF cohort (*n* = 100)
Dysphagia, n (%)	26 (26%)
Type of dysphagia, n (%)	
Oropharyngeal	4 (4%)
Oesophageal	18 (18%)
Pharyngoesophageal	3 (3%)
Severity of Dysphagia, n (%)	
Mild	20 (20%)
Mild to moderate	3 (3%)
Moderate	3 (3%)
Odynophagia, n (%)	56 (56%)
Dysphonia, n (%)	22 (22%)

Remarkable, none of the potential risk factors studied were confirmed in our sample. Neither age, sex, comorbidities nor the BMI, smoking and alcohol consumption were associated with postoperative dysphagia. In the same line, none of the surgical-related factors studied seemed to be associated with postoperative dysphagia, neither surgical duration, side of approach, nor the number of levels operated (Table 3).

**Table 3:** Potential factors associated with the development of postoperative dysphagia

Potential factors	Postoperative dysphagia (*n* = 26)	Normal Postoperative Swallowing (*n* = 74)	*p*-value univariate
Age	59 (13)	53 (11)	0.058
Sex, female	15 (58%)	36 (49%)	0.377
Comorbidities	13 (50%)	29 (39%)	0.264
Previous cervical surgery	5 (19%)	10 (14%)	0.359
Body mass index	27 (5)	26 (5)	0.647
Smoking	8 (31%)	32 (43%)	0.163
Alcohol	1 (4%)	2 (3%)	0.608
No. of cervical levels	2 (1)	1 (1)	0.130
Side of intervention (right)	21 (81%)	65 (88%)	0.407
Surgery duration	116 (45)	125 (50)	0.444
X-ray swelling index	0.75 (0.16)	0.80 (0.21)	0.368
Yoshida score	8.10 (4.34)	7.65 (4.84)	0.704

### Predictive value of radiological scores based on retropharyngeal space oedema

**Fig. 1:**
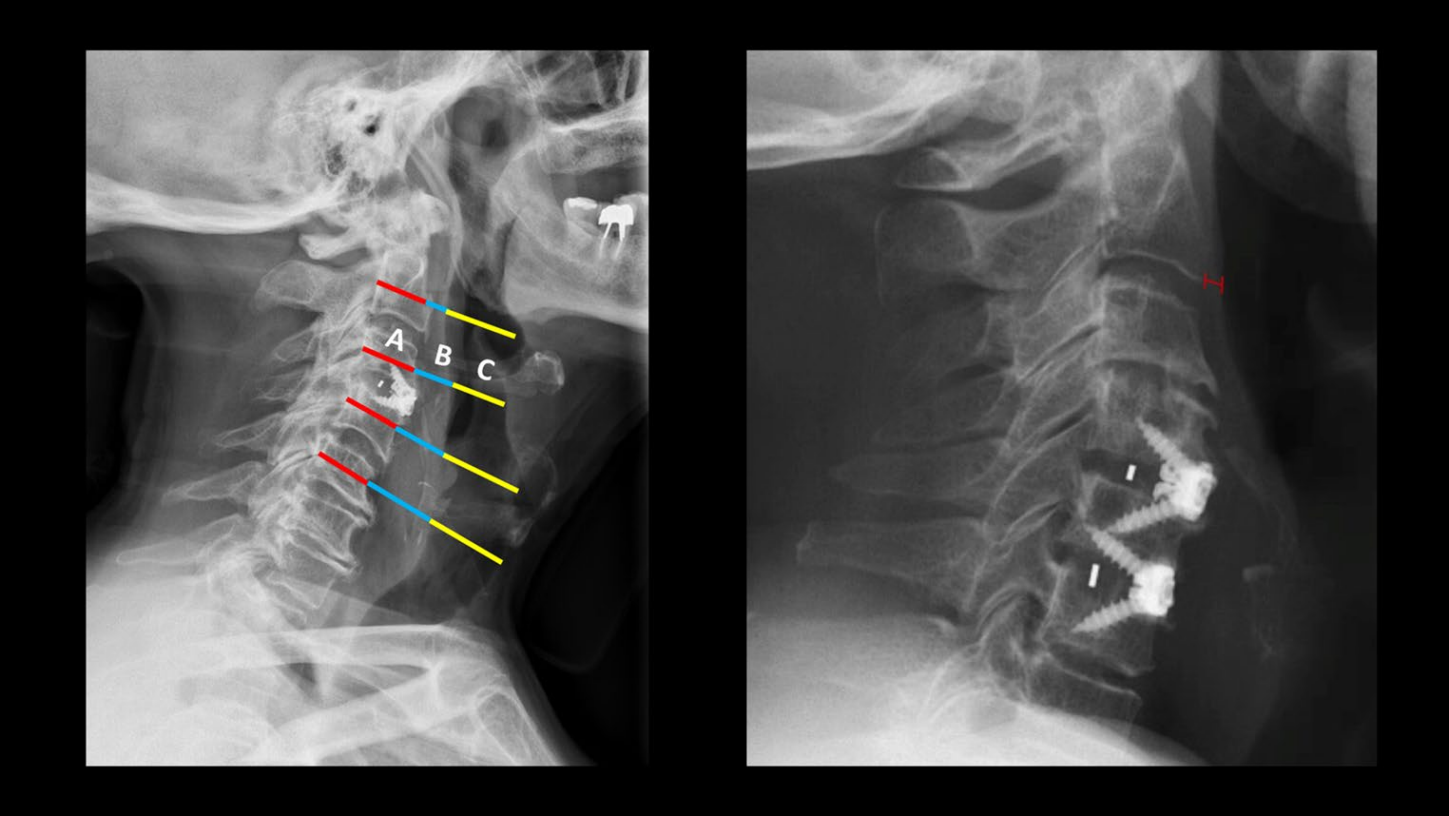
Illustrative example for calculating the radiological indexes based on measuring the retropharyngeal space swelling on the lateral Xray of patients operated for ACDF. *Left*, Haws et al., swelling index was calculated by averaging the ratios of prevertebral swelling to vertebral body diameter at involved ± 1 levels on lateral radiographs, then multiplying by 100. All measurements have been taken in the plane of the mi vertebral body and parallel to the intervertebral disc. The measurements include the AP diameter of the vertebral body (A); the prevertebral soft tissue from the anterior wall of the vertebral body to the posterior tracheal air window (B); and the AP diameter of the tracheal air window (C). *Right*, the Yoshida index was calculated as the anteroposterior distance of the retropharyngeal space measured from the anterior surface of C2 to the posterior tracheal shadow on lateral radiographs

**Fig. 2:**
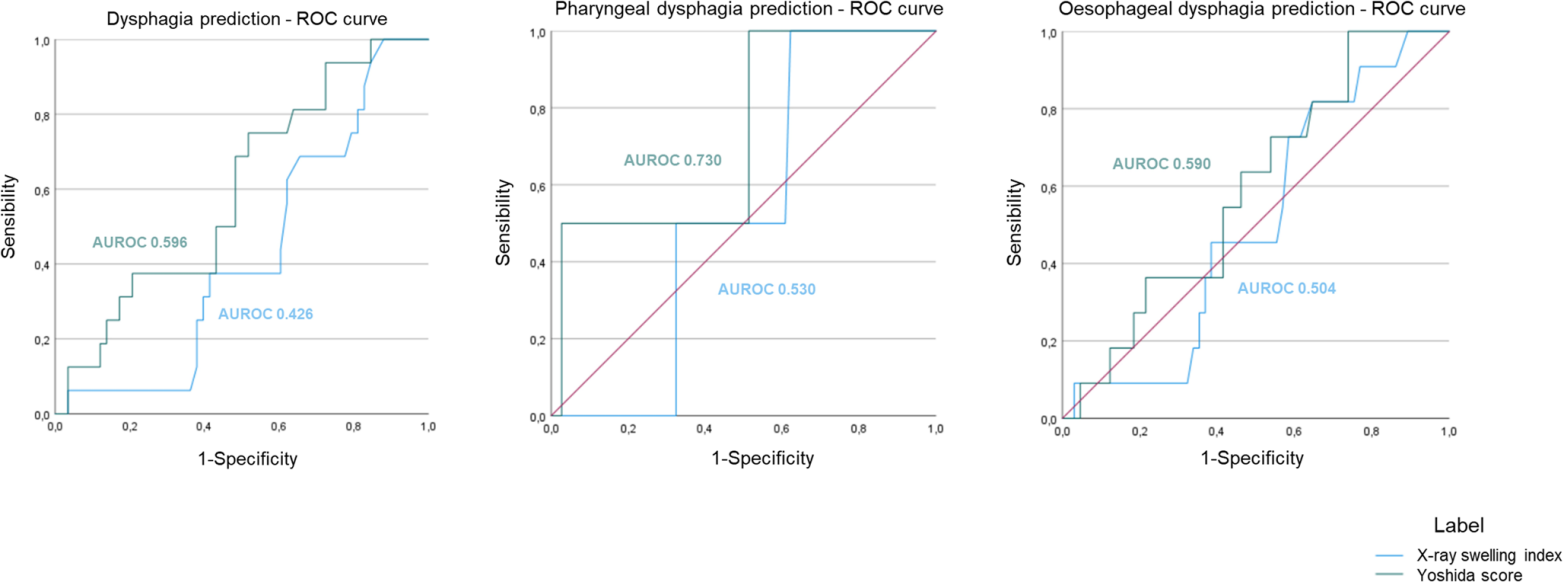
Receiver operating characteristic (ROC) curve demonstrating the capacity of the two indexes (Yoshida and Haws) to predict: any type of dysphagia (*left*), oropharyngeal dysphagia (*middle*), or oesophageal (*right*) dysphagia after ACDF surgery. The area under the curve is given for both indexes under each of the three scenarios

The two radiological scores described in the literature to predict postoperative dysphagia after ACDF were calculated for each patient on a lateral Xray (Fig. 1). Overall, the swelling index yielded an AUROC of 0.426 in our sample to predict dysphagia, while the Yoshida score performed slightly better with an AUROC of 0.596 (Fig. 2).]

Interestingly, when dysphagia was classified according to its anatomical location affected in oropharyngeal, pharyngoesophageal, or oesophageal, the prediction capacity of the two indexes varied. As such, for predicting oropharyngeal dysphagia, Yoshida score yielded an AUROC of 0.730 and the sweeling index an AUROC of 0.530. Conversely, the capacity prediction of both tools was significantly lower in cases of oesophageal dysphagia, with an AUROC of 0.590 and 0.504 respectively (Fig. 2).

## Discussion

In this prospective study, potential risk factors of postoperative dysphagia after ACDF intervention were evaluated. Moreover, the external validation of the two reported predictive indexes of postoperative dysphagia in this scenario was tested. According to our data, none of the patient-related or surgical-related studied factors seem to be significantly associated with postoperative dysphagia. In our sample, the two radiological indexes based on retropharyngeal oedema could have a potential role in predicting oropharyngeal dysphagia, yet limited power in predicting oesophageal dysphagia. These observations could have potential clinical applications and could improve the understanding of the mechanisms causing swallowing deficits in patients undergoing ACDF.

Dysphagia on postoperative day + 1 was found in 26% of our patients, after ad hoc clinical evaluation by a speech and language therapist specializing in swallowing disorders This rate is in line with previous series, however the reported rates vary widely (17–71%) in the literature, depending on the method sued for assessment [7–10]. In the setting of ACDF, postoperative dysphagia has been attributed to multiple factors, including injuries to vagus nerve branches, retraction damage to the oesophagus, or irritation/compression due to the placement of an anterior plate [19–21]. However, none of these factors have consistently been demonstrated as a cause of dysphagia, and swallowing function can be affected even when an anterior plate is not used.

Meanwhile, local oedema in this soft tissue at the retropharyngeal space has been proposed as a mechanism for the swelling disorder after anterior cervical surgery [22, 23]. Based on this principle, two radiological indexes have been described to predict postoperative dysphagia [8, 13]. According to Yoshida et al., the anteroposterior diameter of the retropharyngeal space has a diagnostic value to predict postoperative moderate dysphagia in the acute postoperative stage [13]. In their cohort, the authors stablished a cut-off point of 6.1 mm for the risk of postoperative dysphagia. In our cohort, the Yoshida index seemed to be powered to identify patients at risk of oropharyngeal dysphagia, with a discrimination cut-off point of 0.6, similar to that described in the original publication. The AUROC in the original cohort was 0.700 and in our external cohort was 0.730. However, the same index did not seem to have a good prediction capacity for oesophageal dysphagia (AUROC 0.590). When evaluating the retropharyngeal swelling index described by Haws et al. [8] in our externa cohort, the predictive capacity was poor for both oropharyngeal (AUROC 0.530) and oesophageal (AUROC 0.504) types of dysphagia.

From our data, it may be inferred that oropharyngeal and oesophageal dysphagia might harbour different underlying mechanisms, still not well understood. In fact, in our cohort none of the patient-related or surgical-related factors seemed to be significantly implicated in the development of postoperative dysphagia. This poses a challenge in the preoperative identification of patients at risk, and it remarks the importance of a screening tool in the early postoperative period.

In this sense, the demonstrated external validity of the Yoshida index to predict oropharyngeal dysphagia is a notable finding. Indeed, following the original description and our validation, the use of a simple X-ray may become a routine screening step for patients undergoing ACDF. Meanwhile, identification of oesophageal dysphagia may require further investigation.

Among the limitations of the present study, the limited sample size and the fact that surgical procedures were performed by several different spinal neurosurgeons may impose a bias in the data. Additionally, and because of our hospital protocol and workflow, the cervical X- Ray was made only 6 h after the intervention, being a different timeline than the original authors who calculated the radiological scores to predict post operative dysphagia. This timing may have allowed for identification of later developed retropharyngeal inflammation. Moreover, a long-time follow up of the patients would have given further insight into the evolution of the retropharyngeal oedema and its relation to the resolution of dysphagia; we believe this merits future studies in larger multicentric cohorts. Finally, in terms of dysphagia evaluation, oesophageal dysphagia was classified based on clinical symptoms rather than instrumental confirmation, and the GRBAS scale used to assess dysphonia is perceptual, with known inter- and intra-rater variability, not allowing identification of compensated vocal cord paralysis or aspiration from laryngeal monoplegia. Instrumental evaluations (video fluoroscopic swallow study for dysphagia and laryngoscope assessment for dysphonia) were performed only in patients with persistent symptoms at 6 months, potentially underestimating subtle impairments. Despite these limitations, this approach was chosen for practical reasons: in this prospective study of 100 patients, routine instrumental assessment of all participants was unfeasible. Clinical screening and symptom-based evaluations allowed systematic follow-up, ensured patient safety, and provided meaningful clinical information.

## Conclusion

According to our data, none of the patient-related or surgical-related studied factors seem to be significantly associated with postoperative dysphagia. In our sample, the two radiological indexes based on retropharyngeal oedema could have a potential role in predicting oropharyngeal dysphagia, yet limited power in predicting oesophageal dysphagia. These observations could have potential clinical applications and could improve the understanding of the mechanisms causing swallowing deficits in patients undergoing ACDF. From our data, it may be inferred that oropharyngeal and oesophageal dysphagia might harbour different underlying mechanisms, still not well understood. The use of a simple X-ray may become a routine screening step for patients undergoing ACDF. While it seems robust to predict oropharyngeal dysphagia, identification of oesophageal dysphagia may require further investigation.

## Data Availability

No datasets were generated or analysed during the current study.
